# NHS reference costs: a history and cautionary note

**DOI:** 10.1186/s13561-023-00469-0

**Published:** 2023-11-22

**Authors:** Ben Amies-Cull, Ramon Luengo-Fernandez, Peter Scarborough, Jane Wolstenholme

**Affiliations:** 1 Nuffield Department of Primary Care Health Sciences, Gibson Building, Woodstock Rd, Oxford, OX2 6GG UK; 2 Health Economics Research Centre, Richard Doll Building, University of Oxford, Old Road Campus, Oxford, OX3 7LF UK

**Keywords:** Health economics, Healthcare financing, Healthcare costing

## Abstract

Historically, the NHS did not routinely collect cost data, unlike many countries with private insurance markets. In 1998, for the first time the government mandated NHS trusts to submit estimates of their costs of service, known as reference costs. These have informed a wide range of health economic evaluations and important functions in the health service, such as setting prices.

Reference costs are collected by progressively disaggregating budgets top-down into disease and treatment groups. Despite ongoing improvements to methods and guidance, these submissions continued to suffer a lack of accuracy and comparability, fundamentally undermining their credibility for critical functions.

To overcome these issues, there was a long-held ambition to collect “patient-level” cost data. Patient-level costs are estimated with a combination of disaggregating budgets but also capturing the patient-level “causality of costs” bottom-up in the allocation of resources to patient episodes. These not only aim to capture more of the drivers of costs, but also improve consistency of reporting between providers.

The change in methods may confer improvements to data quality, though judgement is still required and achieving consistency between trusts will take further work. Estimated costs may also change in important ways that may take many years to fully understand. We end on a cautionary note that patient-level cost methods may unlock potential, they alone contribute little to our understanding of the complexities involved with service quality or need, while that potential will require substantial investment to realise. Many healthcare resources cannot be attributed to individual patients so the very notion of “patient-level” costs may be misplaced. High hopes have been put in these new data, though much more work is now necessary to understand their quality, what they show and how their use will impact the system.

## Introduction

The collection of NHS cost data has slowly evolved over time. Accurate cost data was not historically estimated or collected in the NHS as there is no well-developed private insurance market in the UK driving a need for it, unlike countries such as Australia. This resulted in historically weak relationships between need, activity and income [1]. Since 1998, NHS trusts have been required to submit estimates of their costs of providing different services and these were compiled for a wide variety of uses. These ‘reference costs’ have greatly improved our understanding of the economics of the NHS. They are used for a wide variety of important NHS functions, such as exploring the variation in NHS service costs, designing payment systems, setting NHS tariffs (standard remuneration levels for certain activities), understanding non-tariff prices, adjusting local prices, increasing transparency to the public and parliament, planning Programme Budgeting (total amounts to be spent on each service area), identifying the drivers of costs, informing business cases for service development, productivity estimates from the Office for National Statistics and, of course, for a wide range of academic health economic research [2–5]. The breadth and gravity of these functions make data quality a crucial issue [5, 6].

As of 2018/19, NHS reference costs have been progressively phased out and in their place, ‘patient-level’ cost data standards are taking over. Acute trusts moved over to the new methods of cost collection between 2015/16 and 2019/20, followed by ambulance and mental health services in 2019/20. Submissions in July-September 2022 for the financial year 2021/22 finally used the new methods for community services and some previously exempted acute providers, drawing the era of reference costs to an end [7]. Reference costs represented the first serious attempt to understand the origins of costs in the NHS, allowing arcane systems to be updated for the data-driven era [8]. This history of reference costs is rarely discussed, though the story of their development remains essential for understanding data quality and any differences in outcomes that may be found between old and new approaches. Here, we discuss the two broad approaches to cost collections, what the changes imply – and do not imply – for data quality, and the cautions that users of the new data should take. We generally refrain from drawing conclusions about the impacts of these data on the NHS systems that cost collections are used for, as there is very limited ability to extrapolate from methodological changes to what the data may show or their impacts in the system. This paper aims to mark the retirement of reference costs and their important place in the improvement of NHS systems, and highlight attempts to improve NHS costing standards.

## Background to NHS costing

The need to better understand the origins of costs was rooted initially in understanding the basic underlying pattern of demand for healthcare services. Hospital and regional budgets were set in 1948 at the founding of the NHS, when spending was higher in richer areas, and budgets continued to follow the historic pattern. Disparities in budget allocations only widened, so by the 1960s distribution of resources became a political issue. Initially, the *Crossman Formula* was implemented from 1971 to deal with this problem, accounting for local population size, beds and cases. As current utilisation tends to closely follow current resources, this tended to compound the historic pattern so by 1974 its retirement was announced. However, finding a replacement was hard. It was simply not known how resources should be allocated or what benefit changing resource allocations could be expected to have. To address these unknowns, a plan was hatched for an intricate randomised trial for funding health authorities, allocating more resources to some areas, some of which would have those resources earmarked to cardiovascular, cancer or perinatal services. Unsurprisingly, this was not considered politically-acceptable. Instead, the Resource Allocation Working Party (RAWP) was established, eventually developing a funding formula based on Standardised Mortality Rates and disease epidemiology, implemented in 1977. This was successful in reducing the gap in funding between richer and poorer areas, but was particularly unpopular in London where the teaching hospitals lost resources. The contentious formula was maintained until 1989, when a more utilisation-focused approach was implemented after years of increasing emphasis on financial measures of performance [9].

By the 1990s, the acknowledgement of wide variation in total spending had transformed into interest in the related wide variation in cost-per-activity. Research to understand drivers of costs emphasised cost limitation rather than resource allocation. Levers were put in place to attempt to reward more efficient use of resources for both purchasers and providers in the NHS internal market (a landmark healthcare reform creating competition between providers in the NHS) [1, 10, 11]. The ‘Purchaser Efficiency Index’ was introduced as a measure of health authority spending efficiency, using change to weighted activity counts as the numerator and change to budget as the denominator, detecting increases or decreases in activity delivered relative to budget [11]. These attempts to increase efficiency were unfortunately unsuccessful – neither reported costs nor variation reduced [10] and by the end of 1997, the new government agreed these levers had failed to drive efficiency, instead that perverse incentives were leading to fragmentation and unfairness [1, 11, 12]. While much of the infrastructure of the internal market was maintained after 1997, the old measure of commissioner efficiency, the Purchaser Efficiency Index was abolished [10, 11] and new approaches aimed at rewarding performance and collaboration were developed [12]. Reference cost collections began from 1997/98, [10] intending to gain an understanding of baseline efficiency and track improvement (or lack of) [12]. Trusts (NHS providers) were mandated to publish their costs to enable comparison. From these submissions, the NHS Reference Costs Index (RCI) was developed, representing organisation-wide average costs, with similar services grouped as Healthcare Resource Groups, relating to diagnosis, treatment and cost implication [13].

## Reference costs

### What reference costs are

Every year, all NHS trusts in England must submit estimates to the ‘National Cost Collection’ (NCC) of what spending has been undertaken, related to what healthcare activity. These were previously known as reference costs, which NHS England defined as the “average unit cost to the NHS of providing defined services to NHS patients in England in a given financial year”. Although this definition still applies to newer methods of cost estimation, the term itself is generally no longer applied to the dataset, instead being referred to as the National Cost Collection Index (NCCI). Average NHS costs continue to be calculated annually, producing a database of local NHS cost data. This is the richest source available on spending and variations in unit costs across the NHS in England [2, 13]. A reference cost is calculated for each Hospital Resource Group (version 4) (HRG-4) code, defined by the National Casemix Office, which label each patient with a core healthcare activity and features including length of stay, complexity, complications and patient age on the basis of ICD-10 code (International Classification of Diseases version 10, for diagnosis and other clinical features) and OPCS-4 code (Classification of Interventions and Procedures version 4, for medical procedures, formerly the Office for Population Censuses and Surveys classification) [13]. Total costs by activity group, activity count, unit costs of certain services and comparative efficiency between providers can then be assessed.

As mentioned, one of the main purposes of reference costs is to compare the apparent efficiency of different providers. It is not valid to directly compare crude cost per case in a given specialty or activity between trusts, as differences in casemix (that is, differences in diagnosis, treatment approach and clinical complexity) will drive warranted variation that is not related to efficiency [13]. Other justifiable variation in costs may arise from differences in available resources, technology, input costs, difference in priority, service quality, or outcomes. Accounting practices and random volatility may also contribute to variation [10]. To improve comparability, reported costs are aggregated as service-casemix categories called “currencies”. In England, the HRG system groups currencies as “chapters” (specialties) for Admitted Patient Care (APC), outpatient care (OPD) and emergency (A&E) care, as “clusters” (largely by diagnosis group and severity) for Mental Health, “service areas” for community services (relating to the type of service – for example audiology, rehabilitation, Health Visitors), and by level of interaction with the patient for ambulance services [13].

### How reference cost submissions are collected

**Fig. 1:**
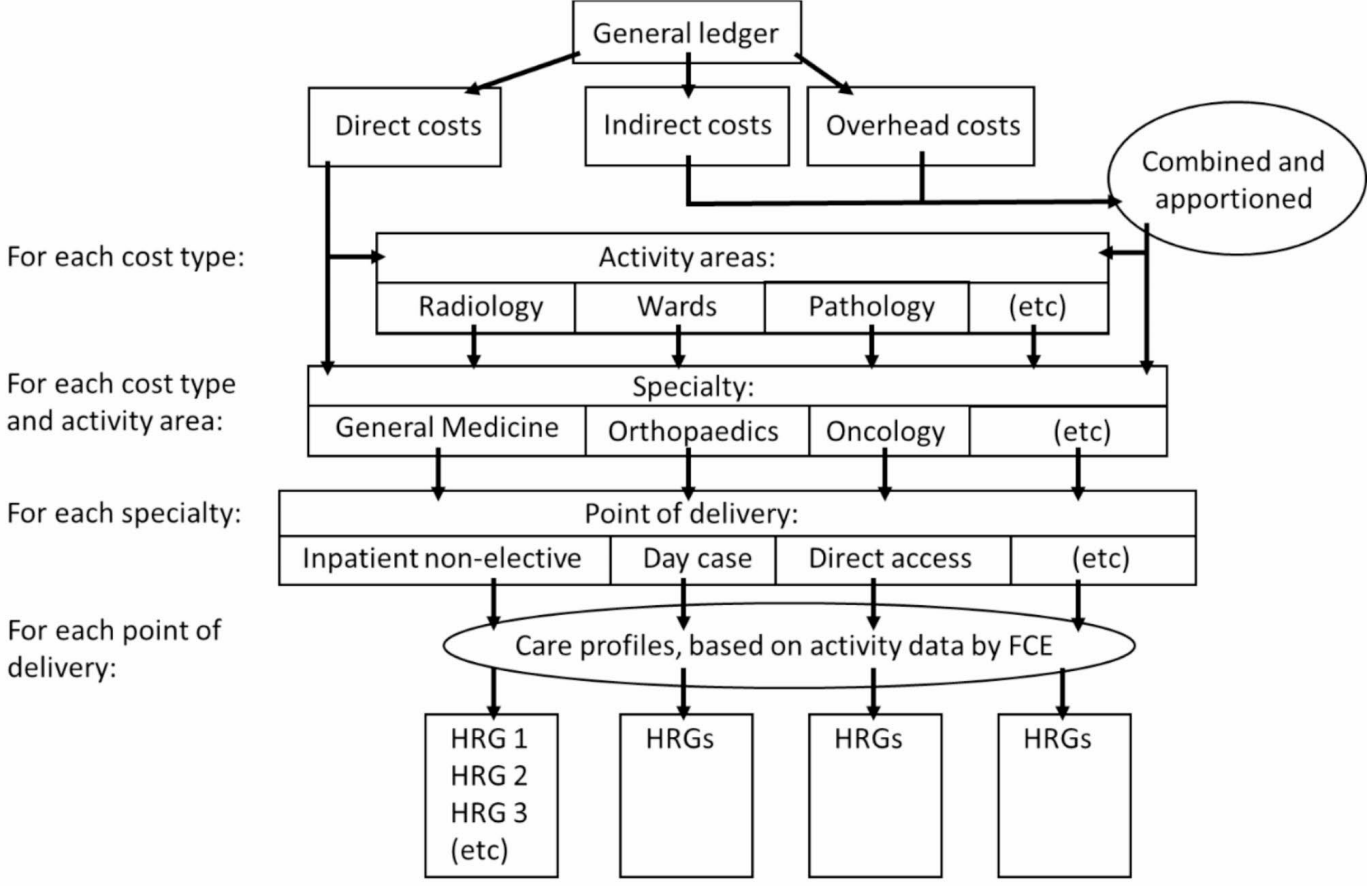
Schematic diagram of the process of calculating NHS reference costs. Adapted from Chartered Institute of Management Accountants (2003) [16] and NHS Costing Manual (2011) [17]

Traditional reference costs have been estimated top-down at the trust level from disaggregated spending pots, on a full absorption basis and associated with the activity that generated it, that is, mutually exclusive and collectively exhaustive of all trust spending necessary for given patient care activity and avoiding cross-subsidisation of services [3, 13]. This process is summarised in figure 1 and as follows. Budgets spent on total direct costs (the requirements of patient care itself e.g. front-line clinical staff), indirect costs (necessary to patient care, but not easily attributable to a given patient, e.g. electricity or bed linen) and overheads (organisational costs not easily attributable to a single patient, such as the Human Resources department) are summarised, [1, 13] then amounts of each of these budget spent on each service line (nationally-standardised specialties) are estimated via different methods. ‘Actual use’ costs are those that can be linked to specific activities, such as surgical devices with fixed costs. ‘Weighted use’ includes factors that require some clinical judgement, such as how much nursing time may be required on average for a group of patients. The amount spent on nursing might be estimated as a factor of the number of nurses on a ward and the need of patients. ‘Apportioned costs’ are those that are divided though crudely, such as the total lighting bill being divided through by the floorspace of each unit [13, 14]. The Healthcare Financial Management Association (HFMA, the professional organisation for UK healthcare finance) published detailed guidance [15] that is approved by NHS England on how to approach these for different types of unit (e.g. medical wards, theatres, mental health units) [13, 14]. Each set of costs is attributed directly to the specialty (which is responsible for each patient) or to activity centres (which consume resources but are not specific to the speciality, such as radiology services). Each trust then aggregates these costs to service lines then apportions costs to point of delivery (inpatient non-elective, outpatient, etc.) and divides down through by amount of patient activity (such as number of bed-days) in that service line, by using coded clinical activity data linking activity to different areas. This gives a crude cost per patient activity, by specialty and point of delivery. A ‘care profile’ is then often used to move from specialty average costs to HRG-specific cost within that service line, to account for what resources those HRG patients consume, such as nursing attention, investigations, theatre time, supported by a series of detailed workbooks in Microsoft Excel attempting to limit variation in practices [1, 13, 16].

### Critique of reference costs

Problems with the first reference costs database quickly became apparent. For example, it was observed in the 2000 release of the dataset that there were gross orders of magnitude difference between providers, for example questioning if the cost of a hip replacement could credibly vary by 20 times between most and least efficient providers, or if the lowest cost submitted (£480) could even pay for the device alone [18]. Limitations of traditional reference cost data quality include variable interpretation of the cost collection guidance inhibiting comparison between providers, substantial exclusions of certain sources of costs from allocation to patient care leading to incomplete data, and the aggregation of costs to HRG averages preventing detailed disaggregation by diagnosis or procedure [8]. No correction for the quality of service was applied, making basic interpretation extremely difficult [19]. Concerns over data quality continued, so in 2009, Monitor (the regulator of NHS trusts financial performance at the time, now part of NHS Improvement) commissioned a major investigation into NHS costing that was published by Lord Carter in 2016 [20]. This was critical of the data quality collected under the reference costs programme, noting there was “huge inconsistency in costing and budgeting approaches … impairing our ability to compare data across the service” [20] and making a series of recommendations.

Alongside the Carter Review, work on how the quality of cost data could be improved was commissioned by the government to Capita and Deloitte. The report from Capita in 2014 audited 50 acute trusts and found financially at-risk trusts were much more likely than others to have inaccurate data submissions: 47% of *at risk* trusts had overall low-quality submissions compared to 20% of randomly audited trusts. Basic errors were being made such as the inappropriate inclusion or exclusion of given activities, double counting impairments, broken or non-transparent software, poor internal processes and scrutiny, inaccurate input activity data and errors in cost allocations. Very few trusts were found to even have appropriately accounted for patient need in nursing costs. They concluded guidance had improved but it was often not being followed, leading to inaccurate reporting [21].

Deloitte undertook a more overarching approach to assessing reference cost quality in 2014, across 178 trusts as part of work on the National Tariff Model (that informs trust remuneration for given activities). They found that one in eight trusts’ submissions 2010-13 contained materially-important errors and that pre-processing of submissions was too basic to account for these (specifically, that the only pre-processing was to remove outliers > 20 times or < 1/20th of the mean). Approximately 50% of HRGs aggregate submissions were non-normally distributed and 30–50% of HRG submissions varied by more than 10% year-on-year. They identified that extreme low unit costs (<£5) were getting less common but that extreme high unit costs (>£50,000) were not. Duplicate costs were a common issue (i.e. exactly the same unit cost given for multiple HRGs) though trusts reporting > 40% duplicate costs dropped from 75 to 40 across the three years (of the 178 trusts). They felt excessive use of judgement rather than following the guidance was leading to erratic performance of a few trusts, for example with 25 trusts having > 50% of their HRG submissions outside the range of 50–150% of mean for that HRG [22].

## Patient-level costing

### The move to patient-level costing

As early as the 1990s, the need to understand the complexity of cost and quality in providing healthcare services was acknowledged. It became to be accepted that while variation was broad it remained lower than in many industries and that simply aiming to drive down cost per unit was simplistic [10, 19].

Patient-level costing was seen as a potential route to develop richer understanding. Calls for using patient-level data for costing had started even by the time the first reference costs were being collected in 1997/98 [8, 10] but problems such as the heterogeneity of systems, difficulty in creating unifying guidelines, problems reconciling the submissions with traditional reference costs and the support that trusts needed to implement the systems all took time to resolve [23].

Monitor proposed mandatory patient-level costing submissions (rather than the traditional top-down approach to costing) in 2012 [24] and the NHS Five Year Forward View 2014 [25] described at length the desire for greater understanding of the detail behind cost drivers. In the context of a frozen cash-terms budget against rising demand, the Five-Year Forward View identified a forecast shortfall in NHS budget of almost £30 billion per year at 2020/21, so an aim was set to limit costs without damaging care. Accurate and detailed costing was seen as essential to understand and continue to improve efficiency [8, 25].

The Care and Quality Commission (regulator of healthcare and social care providers in England) applied further pressure on providers to improve their submissions with its report The State of Health Care and Adult Social Care in England 2014/15. This asserted that producing accurate cost data was a quality benchmark of competent providers [8, 26]. Reform was initiated as Monitor’s Costing Transformation Plan 2015 [7, 27]. This also included new national reporting standards, software requirements, consolidating the programme into one annual submission and a focus on engaging providers in the new standards [27]. Even by the time of this report, in the NCC round 2015/16, 149 of the 237 submissions were already underpinned – at least in some part – by patient-level costing [13]. Providers were given four more years to comply, with mandatory patient-level submissions starting for acute services in 2018/19 then continuing to roll out across other Core services [8].

Cost data quality has gradually improved due to the Carter Report, [20] ongoing work by government and the support of the HFMA [28]. Improvements have been related to developments such as self-assessment checklists, annual updates on standards and targeted external assurance process [13]. In common with Lord Carter, making patient-level reporting mandatory was a key recommendation from both the Capita and Deloitte reports [20–22].

### What patient-level costing is

Patient-level costing is a different approach to estimating activity costs in the NHS. Rather than gradually disaggregating budgets top-down, costs for each patient are estimated based on their individual consumption then those patient pathway costs aggregated to a given level [13, 23].

In the NHS, the systems used to calculate these is referred to as the Patient-Level Indicative Costing System (PLICS) [8]. NHS England defines PLICS as the “IT systems which combine activity, financial and operational data to cost individual episodes of patient care … where an organisation records individual interactions and events that are connected with a patient’s care” [13]. As with reference costs, PLICS takes a full absorption approach of all required inputs and overheads from admission to discharge [13]. PLICS standards “require providers to capture better and more accurate cost information at each stage of a patient’s journey. The data should accurately reflect the ‘causality of costs’ in the system; tracing why costs are being incurred, who is incurring them, by doing what type of activity and for which patient.” [8].

**Fig. 2:**
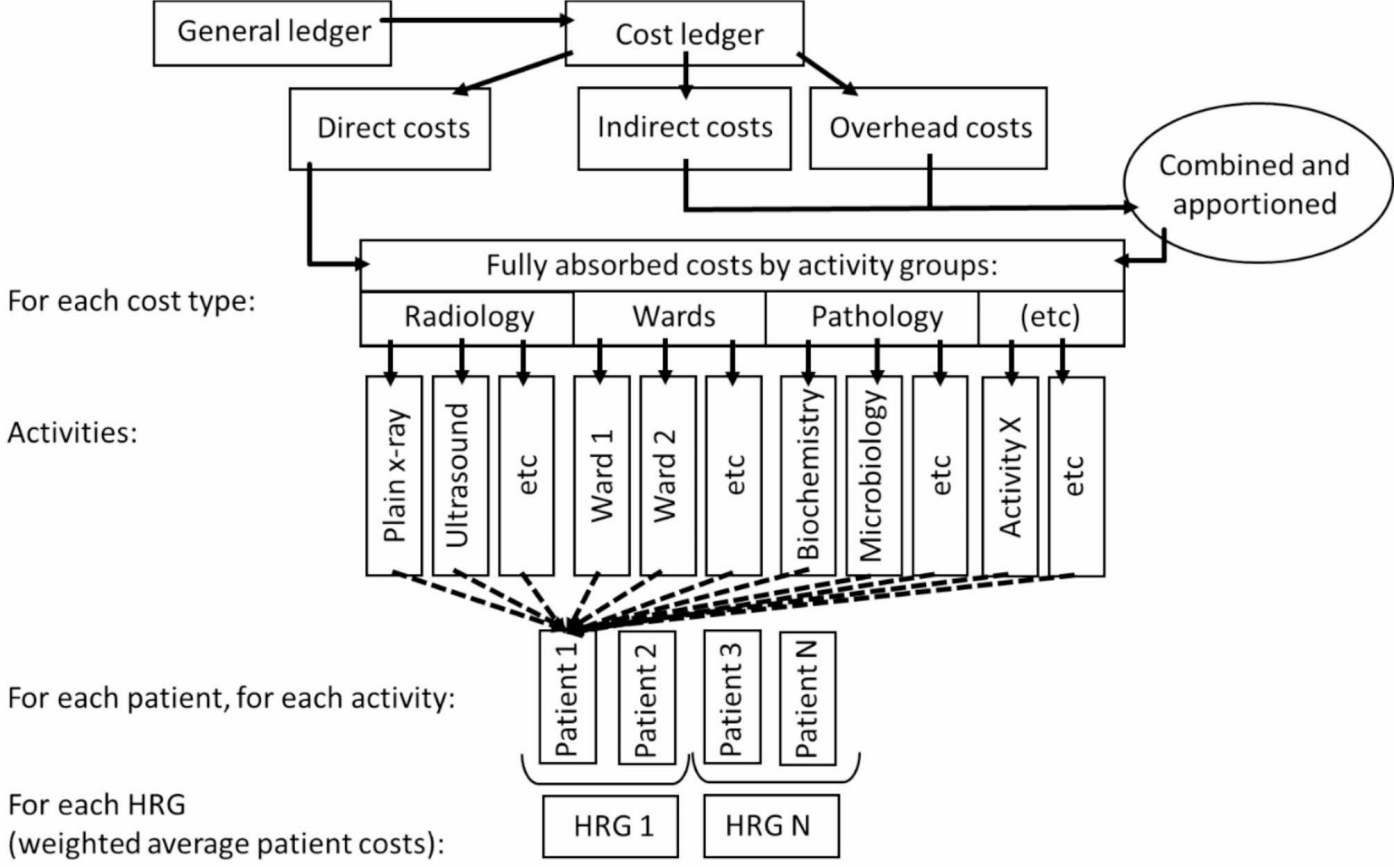
Schematic diagram of the process of calculating NHS patient-level costs. Adapted from HFMA Acute Care Costing Guidance 2016/17 [15] and Gray 2016 [29]

To support the consistency that makes PLICS valuable, NHS England have published extensive guidance covering each type of service provided [14, 28, 29]. Five main Standards act as the broad framework, summarised in figure 2. They start with 1, ensuring the *General Ledger* (outlay) is defined and structured appropriately, then 2, disaggregating and reaggregating outlays into predefined clinically-relevant categories (the *Cost ledger*), for example aggregating wages, National Insurance and pensions into ‘staff costs’ then disaggregating them by role. Standard 3 details how to map the Cost Ledger as direct costs or overheads and link these *resources* (e.g. staff, equipment, consumables) to activity groups (e.g. theatres, radiology) then to specific cost-generating *activities* (e.g. a particular operation or test) using *relative weight values* (e.g. what proportion of a member of staff’s time is spent on given work). Standard 4, assigning these resources to specific patients (*matching*) is a critical step, [14, 29] that is, the clinically-appropriate association of costs to patient episodes, relating to the causality of costs. This is informed by extensive rules and detailed activity data known as ‘feeds’ that quantify how much resource is used by patient episode. Costs are aggregated by patient, then average patient costs are calculated within each HRG. The fifth Standard is that aggregated costs then must be reconciled against accounts to check they remain consistent. Further Standards [6–10] relate to other factors such as audit and self-assessment of reporting quality. These standards not only change the approach but also aim to greatly improve consistency of approach between providers [14, 15, 28, 29]. The broad difference from reference costs methods is that for reference costs, spending is progressively disaggregated down to the unit level then differences between HRG within each specialty are estimated based on judgement, while for patient-level costs are disaggregated down to activities that consume resources, then aggregated by patient based on data. This process is is different in important ways, replacing judgement with data – specifically at the point of the care profile.

Although moving the point of aggregation of costs to that of patient consumption is a valuable improvement in tracing the flow of costs, it’s important to bear in mind that very little activity actually exerts a cost on an individual basis – the unit costs matched to patients are calculated as average costs of producing that unit [30]. This is partly acknowledged in separating direct costs from indirect and overhead costs, though even within direct costs the reality is difficult to reconcile with the intention for “patient-level” costs, for example, allocating staff time to various activities is challenging. Doctor or nurse time spent for patients in each HRG (let alone for each patient) cannot feasibly by measured, so must still be based on judgement and averages. In places, therefore, judgement in the reference cost process has not been replaced with data but simply moved to a different point, specifically in the estimation of unit costs of some patient activities. Therefore, the notion of patient-level spending may be appealing but ultimately not met, so more descriptive terminology here would seem appropriate – for example, simply *patient-level aggregate unit costing* would be more intellectually honest.

However, the extensive reform of the process also offers other value, particularly in providing an opportunity to create greater consistency between trusts, both in terms of interpretation and adherence. That costs are simply being synthesised in consistent ways may be the greatest contribution to data quality, so it is essential to remember that PLICS do not necessarily improve consistency if the guidance isn’t appropriately followed. Our difficult experience attempting to progressively improve the data quality of reference costs indicates it will be essential to prevent the embedding of poor practice that could attenuate future improvement.

### Potential benefits and risks from patient-level costing

Groundwork by NHS Improvement in 2015 identified that some trusts that were already implementing PLICS had achieved large benefits from small cost investments [8]. Examples included identification of mis-coded procedures leading to lost income of £160,000 per year, omission of claims for Cancer Drugs Fund income, and more efficient running of ophthalmology theatres to the order of £211,000 per year. Potential benefits included increased detail, improved comparability, identification of areas of inefficiency, increased long-term stability of providers, and informing local commissioning and tariff variation. It was felt other non-cost benefits were also achieved, such as increased clinician engagement with finances and cost implications of different choices [8].

There are also potential downsides. Interestingly, early uses of PLICS increased the risk of inaccurate reporting. In Capita’s 2014 report, PLICS were felt to add complexity and bureaucratic burden. It was also realistic that “Non-admitted patient care services still present the same challenges to cost with or without PLICS” [21]. It was acknowledged that larger, broader and more complex providers with better upstream cost systems would find it more expensive to implement PLICS and that any successful implementation relied on good senior leadership and buy-in across management and clinicians, not only for implementation but for the ongoing embedding of cost data in decision-making, requiring a complex managerial, financial and clinical skill-mix that may be difficult to access [8]. Despite the more sophisticated approach, there is still no correction for the service quality, as with reference costs [19].

It remains to be seen what the impact of the new methods will be, with even basic differences from reference cost submissions being poorly understood. As the approach is different and the aim is to improve data accuracy and comparability, many trusts’ submissions may materially change, altering how we understand between-trust variation or even average costs for given services. Assuming the quality of newer releases is superior, these data may illuminate potentially deleterious historic impacts of the previous cost methods on the configuration of the NHS [5, 6]. These new data will provide a greater ability to understand the drivers of costs moving forward. It is crucial to observe, to avoid misinterpretation, that these new methods do not differ materially in what they intend to represent – neither dimensions of service quality nor need can be inferred, while both of these factors may confound the appropriate understanding of the data [1, 11].

### The cautionary note - what PLICS are and are not

There are key differences in approach between reference costs and PLICS laid out above, specifically that disaggregation is to the level of the healthcare activity for PLICS, followed by aggregation to the patient and HRG level, while for reference costs, budgets are progressively disaggregated to the HRG. There is less judgement involved in PLICS due most importantly to the replacing of ‘care pathways’ in the reference costs process with activity data feeds. This should lead to better attribution of costs to patients and HRGs, better reflecting the reality of the exertion of costs. However, adherence to guidance and consistency in approach are not guaranteed. Indeed, that the early use of PLICS was associated with greater risk of errors [31] implies that close monitoring will be critical, to allow learning to be gained and disseminated effectively.

Intentions exist that they will reveal a causality of costs and differences in crude costs of activity, though answering this will be the subject of a large body of empirical work, requiring the separation of efficiency from other sources of variation. The legitimate sources of variation mentioned above provide major cause for concern that PLICS could offer available answers.

Ultimately, they should provide an improved means to an end, but much more local and national investment will be needed. Firstly in good implementation, identifying good/ poor practice, spreading this learning, and supporting poor-performing trusts to improve reporting standards, and secondly into understanding what they mean, both in terms of how costs arise in the NHS but also what the implications of old data was versus new – on the assumption these new data are more consistent with one another and correlate more closely to reality. Data monitoring will be an important piece of this – including tracking duplicate unit costs, extreme values and undue within-unit variation along with many other data quality markers.

As has been commented many times in the past, understanding the causality of costs is no simple thing. It may be highly granular to the point of sub-HRG-level approach to patient care with nuance around factors that don’t particularly pertain to patients’ clinical factors or treatment choices, such as how well a team communicates on a specific ward or how much help patients’ families are able to provide in an area. Only once differences in reporting are known to be minimised and justifiable variability in apparent costs understood, can other work start to address less legitimate differences in costs (e.g. inefficiency).

For HEE, certain flexibility and granularity may be possible, so once data quality are better understood, the ability to rely on better data may facilitate broader use and more robust evaluations. Data linkage also offers big opportunities. For example, broader uses of whole-pathway HEE may be facilitated by linkage as the patient’s outcomes could be linked to their costs and followed through the system. Although the costs themselves are patient-level within a specific HRG code treatment event, linkage could allow them to be seen as part of a bigger picture with a patient’s other hospital services demand and primary care usage, for related and unrelated healthcare needs. Alternately, dimensions of clinical quality could be linked to costs, such as more expensive pathways conferring better outcomes, or separating treatments from their complications. It may become possible to better understand where the costs of complications or multimorbidity are additive, multiplicative or otherwise, for whom and in what situations. There is a risk that HEE relying on old reference costs may yield very different results if repeated using PLICS simply due to previously inaccurate data. Work identifying the higher risk areas of reference cost data would help NICE to better understand where their Health Technology Assessments may be at greater risk of being affected.

## Conclusions

Having previously no routinely-collected cost data, NHS reference costs have been essential for advancing our understanding of NHS economics. Methods for understanding need and for allocating budgets were crudely based on pre-existing supply, so the introduction of reference cost data to the process has been an important step. Data quality has been an issue since the beginning of the Reference Costs Index, with repeated critique of the accuracy of individual submissions, consistency of approach, crude data cleaning and excessive reliance on judgement in the process. By 2009, change was instigated and the Costing Transformation Programme [27] formally recommended replacing the previous approach with patient-level methods in 2015, supported, shortly after, by the Carter Report [20]. Trusts were given four years to comply with new guidance under the Costing Transformation Plan and reference costs were finally phased out in 2021 [7]. The costing process is improved in important places, replacing judgement with data. The new data remain poorly understood and a great deal of work will be required to understand them, along with their impacts on the health economy and our understanding of the drivers of costs. What we find may be materially different from what we found from reference costs, with important implications. While data quality may be better, this cannot be assumed and poor practice must be identified and learned from, then trusts supported to improve. More research will be required in the health service to better understand the dimensions of quality as they relate to efficiency and how the relationships between quality, cost and overall efficiency are understood.

## Data Availability

Literature was identified from government websites and peer reviewed papers were sourced through the institutional library.

## Declarations

### Authors’ information

BAC is a clinical academic in non-communicable disease modelling and health economics, focusing on prevention of obesity-related diseases. RFL is a Associate Professor of Health Economics working mainly in the healthcare cost and economic effects of cardiovascular diseases. PS is Professor of Population Health, working on evaluations of population approaches to increase the uptake of healthy, sustainable diets across a wide range of approaches in the food system. JW is Associate Professor of Health Economics with over 25 years of experience in conducting economic evaluations for health policy makers and exploring methodological issues relating to the design and analysis of economic evaluations.

## Abbreviations

A&E: Accident and Emergency
APC: Admitted Patient Care
HFMA: Healthcare Financial Management Association
HRG-4: Healthcare Resource Groups version 4
ICD-10: International Classification of Diseases version 10
NCC: National Cost Collection
NCCI: National Cost Collection Index
OPCS-4: Office for Population Censuses and Surveys
OPD: Outpatients Department
PLICS: Patient-Level Indicative Costing System
RAWP: Resource Allocation Working Party
RCI: Reference Costs Index
